# Real-Time Monitoring of Protein Conformational Changes Using a Nano-Mechanical Sensor

**DOI:** 10.1371/journal.pone.0103674

**Published:** 2014-07-31

**Authors:** Livan Alonso-Sarduy, Paolo De Los Rios, Fabrizio Benedetti, Dusan Vobornik, Giovanni Dietler, Sandor Kasas, Giovanni Longo

**Affiliations:** 1 Laboratory of Physics of Living Matter, Institute of Physics of Biological Systems, School of Basic Sciences, École polytechnique fédérale de Lausanne, Lausanne, Switzerland; 2 Laboratory of Statistical Biophysics, Institute of Theoretical Physics, School of Basic Sciences, École polytechnique fédérale de Lausanne, Lausanne, Switzerland; 3 Faculty of Biology and Medicine, Department of Fundamental Neurosciences, Lausanne University, Lausanne, Switzerland; 4 Istituto Superiore di Sanità, Rome, Italy; LAAS-CNRS, France

## Abstract

Proteins can switch between different conformations in response to stimuli, such as pH or temperature variations, or to the binding of ligands. Such plasticity and its kinetics can have a crucial functional role, and their characterization has taken center stage in protein research. As an example, Topoisomerases are particularly interesting enzymes capable of managing tangled and supercoiled double-stranded DNA, thus facilitating many physiological processes. In this work, we describe the use of a cantilever-based nanomotion sensor to characterize the dynamics of human topoisomerase II (Topo II) enzymes and their response to different kinds of ligands, such as ATP, which enhance the conformational dynamics. The sensitivity and time resolution of this sensor allow determining quantitatively the correlation between the ATP concentration and the rate of Topo II conformational changes. Furthermore, we show how to rationalize the experimental results in a comprehensive model that takes into account both the physics of the cantilever and the dynamics of the ATPase cycle of the enzyme, shedding light on the kinetics of the process. Finally, we study the effect of aclarubicin, an anticancer drug, demonstrating that it affects directly the Topo II molecule inhibiting its conformational changes. These results pave the way to a new way of studying the intrinsic dynamics of proteins and of protein complexes allowing new applications ranging from fundamental proteomics to drug discovery and development and possibly to clinical practice.

## Introduction

Monitoring protein activity is of paramount importance in several domains of biology and medicine, such as proteomics [1], [2], investigation of biomolecular interactions [3], [4] or drug development [5]. Proteins can switch between different conformations in response to stimuli, such as pH, temperature variations, or binding of ligands. Such plasticity and its kinetics have a crucial functional role, and their characterization has taken the centre stage in protein research [1], [6], [7]. For example, human Topoisomerase type II (Topo II) is a particularly interesting enzyme capable, through the hydrolysis of ATP, of managing tangled and supercoiled double-stranded DNA by changing its topology, thus facilitating numerous physiological processes such as gene expression, cell division, transcription or duplication [8]. Because of this it is often employed as target for anticancer drugs [9] and disparate techniques have been used to characterize its conformational changes [10]–[12]. Presently, several tools are capable of detecting protein-ligand interactions (*e.g.* protein-binding microarrays [13], [14]) and of measuring the rates of the associated transitions (*e.g.* optical tweezers [15] and Fluorescence Resonance Energy Transfer [16], [17]). Unfortunately, most of them involve sizeable setups and complex biochemical preparations. This hampers their scalability, introduces greater complications in the assessment of the intrinsic kinetics of proteins and of how they are affected by different environmental conditions and ligands, *e.g.* drugs acting at the molecular level.

We have very recently introduced a novel technique, the nanomotion detector, based on the well-established nanomechanical sensor technology [18]. This technique is capable of measuring movement at the nanoscale and we have used it to characterize, with unprecedented speed and sensitivity, the metabolism of living systems [19], [20]. In this work, we exploit this diagnostic tool to characterize the dynamic properties of Topoisomerases, by studying their interactions with ATP and the consequent conformational changes and investigating the timing of the transitions.

Although possibly not as accurate, nanomechanical sensors represent a viable, sensitive, parallelizable and down-sizeable alternative to more conventional techniques. Among them, Atomic Force Microscope (AFM) microcantilever sensors are now routinely used to study ligand-receptor interactions [21], [22]. Due to their sensitivity and their large dynamical range, cantilever sensors have the potential to provide a breakthrough in the investigation and characterization of biological systems [23] including conformational changes in proteins [24] and, in particular, the ATP hydrolysis in enzymes [25]. However, up to now, such capabilities have not been exploited to investigate in detail the dynamics of these conformational changes, restricting the focus on static effects [26], [27]. To characterize with higher detail the dynamic properties of specimens, we have developed a new system that measures the low frequency fluctuations of a nanomechanical sensor (<1 kHz), thus circumventing the major limitations of the currently available systems. This technique is remarkably sensitive and can detect sub-Ångstrom motions in physiological media.

In this study, we report the use of cantilevers as sensors to investigate the ATP-induced conformational changes of Topo II, focusing in particular on the correlation between the ATP concentration and the resulting fluctuations of the sensor.

## Materials and Methods

### Substrates, enzymes and reagents

Human Topo II α p170, ATP and aclarubicin were purchased from TopoGEN. Magnesium chloride hexahydrate, sodium chloride, Caged-ATP, EDTA, APTES, DTT and AMPPNP, all with analytical grade, were supplied by Sigma-Aldrich. All complexes were prepared in tubes prior to deposition or injection.

### The working buffer

To perform the measurements we chose to work with a buffer (working buffer) that allows a good attachment of the Topo II while leaving the freedom of movement to the enzymes allowing them to undergo conformational changes. The [Mg^2+^]/[Na^+^] ratio is crucial for the establishment of optimal conditions [28], so we used a solution containing 50 mM Tris-HCl (pH 8.0), 150 mM NaCl, 10 mM MgCl_2_ and 0.5 mM DTT. This working buffer produces an adsorption of the enzymes to the surface while allowing them, at the same time, to undergo conformational changes [29], [30].

### Topo II–DNA complex preparation and cantilever functionalization

Human Topo II (107.6 nM) was incubated with negatively supercoiled pBR322 DNA (200 nM), for 30 min at 30°C, in the working buffer. All Topo II–DNA complexes were prepared in tubes and immediately applied to the functionalized cantilevers for measurement.

### Brief description of the technique

The time-dependent fluctuations of the sensor were recorded through a dedicated electronic system. The measurements were performed exploiting the laser-based transduction system of a Multimode AFM (Bruker, Santa Barbara, CA). This microscope was equipped with a Nanoscope IV controller, a PicoForce Control Module and a liquid fluid cell. The microscope was isolated from the environmental low-frequency vibrations using a Halcyonics i4 anti-vibration table (Accurion, Goettingen, DE) and isolating the entire microscope from external noise with a closed hood.

The cantilever deflection data was collected using a data acquisition board (National Instruments, USB-6211) and a LabVIEW software (National Instruments). Since the typical timescale of Topo II conformational changes is in the order of the milliseconds [31], the cantilever fluctuations were recorded with an adequate temporal resolution (a sampling frequency of 100 kHz was chosen). The deflection was converted in nanometers by measuring the cantilever’s deflection calibration factor.

The measurements were performed while different solutions were flowed in the analysis chamber. For instance, media containing increasing concentrations of ATP were flowed at a constant rate and for minimally one minute. After this time period, the next solution (with a different ATP concentration) was injected in the system.

Similar approaches are present in literature, using conventional AFM setups to investigate biological systems, including full cells [32], [33] or proteins [24], [34]. These works are remarkably similar to our system and demonstrate that a cantilever sensor can be used to investigate the conformational changes in proteins. Thus, a nanomotion detector setup can be used to monitor and study the ATP hydrolysis by Topo II.

The raw, unfiltered data were analysed using Mathematica (Wolfram). The algorithms were developed to analyse the deflection signal, calculate its variance and the power spectral density (PSD). The deflection as a function of time was recorded and the variance (*Var*) function used to characterize the deflection fluctuations (*Z*_i_) was calculated using following formula:
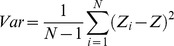



The Fourier analysis was used to calculate the spectral estimate of the deflection data.

Data is presented as mean value ± sd. The Kruskal-Wallis test, followed by Dunn’s multiple comparison test, was used to compare three groups (P<0.05 was considered significant).

### Immobilization of the Topo II and Topo II-DNA complexes on the sensor

Triangular silicon nitride cantilevers (DNP-10, Bruker, Santa Barbara, CA) with a 225 µm length and a spring constant of 0.01–0.04 N/m were used. In order to allow the immobilization of the Topo II molecules, the cantilevers were functionalized using APTES (3-aminopropyltriethoxysilane), a highly effective silane coupling agent used on a wide series of substrates to enhance molecule and cell adhesion. The cantilevers were prepared by immersing them in a 0.1% water solution of APTES for 1 min, then rinsing thoroughly with ultra-pure water and drying using a gentle flow of air. The APTES-functionalized cantilevers were immersed for 15–30 min at room temperature either in the Topo II (107.6 nM and 53.8 nM were chosen for the experiments described here) or in the Topo II-DNA complex solution. The cantilevers were then rinsed with the working buffer and placed into the analysis chamber equipped with inlet and outlet tubes to allow the flushing of different solutions through a low-noise liquid injection system [35].

### Injection system

Different imaging buffers were assayed using a gravity injection system based on the one published by Thomson *et al.* [29]. To ensure a complete exchange of the fluid, 400 µl of each solution were injected at a flow rate of 0.1–8 µl/s. This injection system allows exchanging the fluid several times in a single experiment, and thus the chemical environment in which the deflection measurement is being conducted. In the presented data such system was employed to flow into the liquid cell, separately or successively, the working buffer, the ATP solution (0.2 µM–2 mM), the AMPPNP solution (0.2 µM–2 mM), or the aclarubicin solution (100 µM).

### Temperature control

The temperature control is a crucial parameter when performing experiments on enzymes, since their activity is extremely temperature-dependent. Furthermore, cantilevers as nanomechanical sensors are extremely sensitive to temperature variations.

In view of the importance of this parameter, we controlled the temperature by placing the microscope in a temperature-controlled chamber and all the solutions were left to thermalize for minimally 1 hour prior to starting the experiments. The temperature of all the injected media was controlled just before the injection using a bimetallic temperature sensor (DT120, Rüeger, CH). This ensures that the temperature throughout the entire experimental analysis is constant within 0.1°C. Before starting any acquisition we waited for the system to stabilize; less than 30 minutes were needed to obtain a perfectly stable cantilever. Any effect of local laser heating or cantilever static bending was, at that point, negligible.We chose to work at room temperature to ensure the stability of the enzyme throughout the entire experiment. Indeed, Topo II molecules are known to have a lifetime at 37°C of around 1 hour. By working at 20°C, we expect the lifetime of the enzyme to be sizably larger than 1 h, thus much larger than the typical experimental time-scale.

## Results

**Figure 1:**
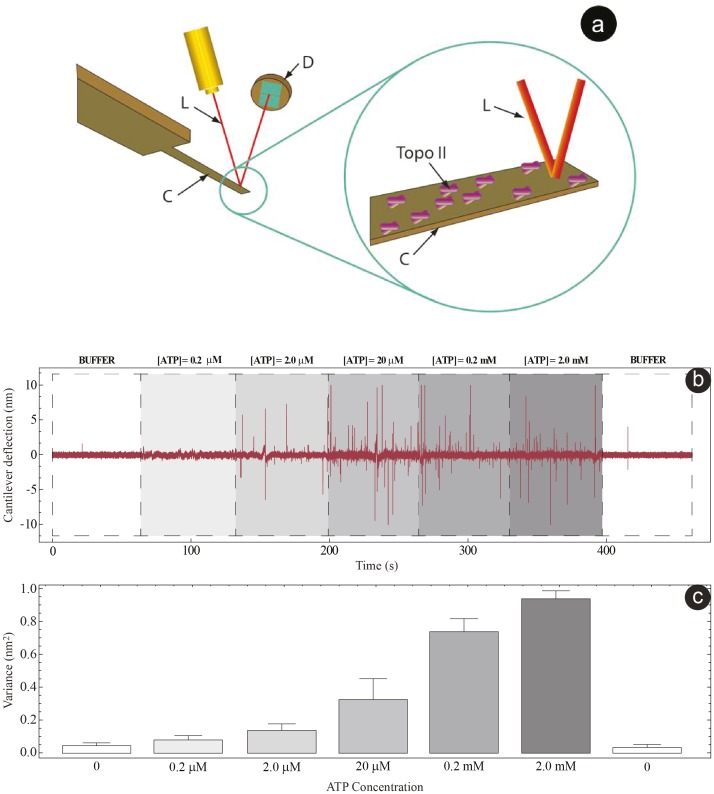
The experimental setup and the experiment using high Topo II concentration (107.6 nM). Panel a: Schematic illustration of the experimental setup: C) cantilever, L) laser beam, D) photodiode. Topo II indicates the molecules adsorbed to both sides of the cantilever. Panel b: The cantilever deflections as a function of ATP concentration. Different media were flowed through the analysis chamber: buffer (with no ATP), ATP-enriched medium, containing in order 0.2 µM, 2.0 µM, 0.02 mM, 0.2 mM and 2.0 mM ATP, and then again the no-ATP buffer. Panel c: Corresponding variance values.

The experimental setup used in these experiments is sketched in Figure 1a and is described in detail in File S1. We functionalized a commercial AFM cantilever and exposed it to a solution containing Topo II (107.6 nM). This resulted in a uniform enzyme coating of both sides of the cantilever (more than 10^7^ molecules on the entire cantilever, for more details on the methods and on the characterization of the sensor see Figures S1, S2 and S3 and the discussion in File S1). In previous experiments, some of us have demonstrated that Topo II molecules, in these conditions, are active and that they maintain their enzyme functionality on DNA, as well as their basal activity [30]. We then introduced it in the analysis chamber and its fluctuations were collected as a function of time. Several solutions containing increasing concentrations of ATP were consecutively flowed through the chamber at a rate of 4 µl/s. Figure 1b shows seven successive measurements of the fluctuations of the lever as a function of time, the first and last corresponding to the buffer without ATP, while the others to media enriched with increasing concentrations of ATP. The variance of the fluctuations was calculated by dividing the data in 10-second chunks and is plotted in Figure 1c. The variance increased with the concentration of ATP and returned to low levels when the ATP was flushed out of the chamber. These results are in excellent agreement with similar experiments performed using conventional biochemical techniques. In absence of DNA, the basal ATPase rates of Topo II are less than one order of magnitude lower than the DNA-stimulated rates and follow a standard Michaelis-Menten kinetic model [36].

The experiment was repeated with cantilevers functionalized with a solution that had half the concentration of Topo II (53.8 nM) (Figure S4 in File S1). This resulted in an overall decrease of the variance of the deflections that maintained nonetheless the same dependence on the ATP concentration.

In an effort to better characterize the physical phenomenon that caused the fluctuations we measured, we studied the evolution of Topo II when exposed to Caged-ATP, a photo-activated ATP that requires exposure to UV light to undergo hydrolysis. The introduction of Caged-ATP in the analysis chamber appeared not to influence the system, which instead exhibited enhanced fluctuations immediately after the illumination with UV light (Figure S5 in File S1). Since the physical and optical properties of the medium before and after the exposure to the UV light are identical, this rules out any interpretation of the origin of the fluctuations that does not involve the Topo II hydrolysis of the ATP.

This experiment and the direct correlation between the variance of the fluctuations of the sensor and the concentrations of ATP and Topo II are clear indications that the sensor had indeed measured the activity of the molecules present on its surface. Moreover, the entire process was reversible, as was demonstrated by changing the medium back to the working buffer at the end of the experimental cycles and finding the same variance of the fluctuations as at the beginning of the cycle, before the injection of ATP.

To support an interpretation of the collected data we performed control experiments aimed to disentangle the effect of the ATP hydrolysis and of the enzyme conformational change. First, we used a non-hydrolysable ATP-analogue (AMPPNP) in place of ATP. AMPPNP dynamically binds to the ATPase regions of the Topo II, inducing a conformational change and, since it cannot be hydrolysed, it is later released back to the solution [10]. Indeed, no energy is used in this process and this is clearly indicated by the observed variance of the deflections, which is not influenced by the presence of AMPPNP at any tested concentration (see discussion and Figure S6 in File S1). Next, we immobilized apyrase on the cantilever, an ATPase that is much smaller than Topo II. As for the Topo II we studied the response of the cantilever when we exposed it to different ATP concentrations (see discussion and Figure S7 in File S1). These experiments and all the other controls we carried out to exclude any physical or optical artefact (Figure S8 in File S1 summarizes these control experiments) all point to the same interpretation of the origin of the measured fluctuations. The fluctuations of the Topo II coated cantilevers exposed to ATP are a clear hallmark of an activated process driven by the energy from ATP hydrolysis and not of the hydrolysis itself. Furthermore, the high signal acquisition rate allows a much more detailed characterization of the dynamical properties of the sensor fluctuations, thus obtaining accurate information on the temporal behaviour of the proteins. In particular, the intrinsic stochastic nature of the detected processes implies that the relevant time scales of the phenomena are better described by the autocorrelation function of the signal or, alternatively, by its Fourier transform, namely the power spectral density (PSD).

**Figure 2:**
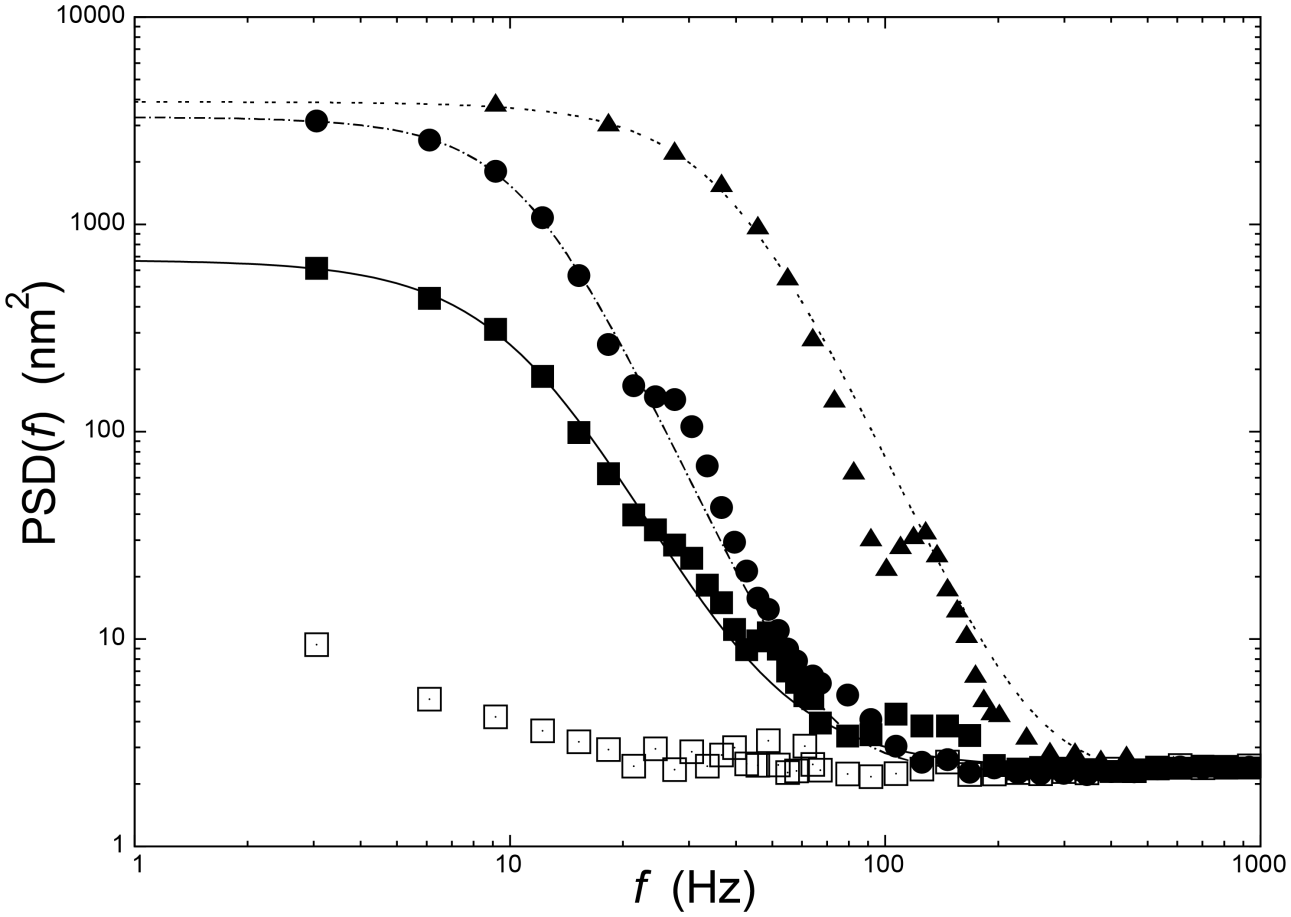
The model analysis. Power spectral density plot of the deflection data for different ATP concentrations (2.0 µM – black squares, 0.2 mM – black circles and 2.0 mM – black triangles) for a 107.7 nM Topo II preparation concentration. The white squares indicate the buffer data that were used as baseline. The data for the 2.0 mM concentration were shifted horizontally (by a multiplicative factor of 3) to enhance readability. For each curve the corresponding fit from the model is shown.

Without ATP, the fluctuations of the cantilever are driven by mere thermal noise, whose PSD (white squares in Figure 2), was used as a baseline for the analysis of the other cases.

As the ATP concentration increased, the low-frequency part of the spectrum (below 100 Hz) raised correspondingly from the baseline (black squares, circles and triangles in Figure 2), indicating that the process transduced by the sensor is not entirely thermal, but also driven by the energy from the hydrolysis of ATP. Moreover, time-scales in the millisecond range are compatible with the known rates for the conformational changes of Topo II in presence of DNA [17]. As discussed in detail in File S1, the difference in the complete cycle turnover rate measured in the presence or absence of bound DNA is typically less than one order of magnitude and strongly dependent on the environmental conditions. As a consequence, the rates of the different substeps of the cycle will be comparable with or without DNA [36].

Previous studies have demonstrated that Topo II undergoes cycles of ATP binding; ATP hydrolysis and ADP release, which produce conformational transitions (see File S1). Thus, a three-state model was chosen to describe the Topo II system, where each of the three transitions is characterized by specific rates: an empty *apo* state (1); an ATP-bound state (2) and an ADP-bound state (3) (Figure S12 in File S1). The *apo* state converts into the ATP-bound state by ATP binding; the ATP-bound state turns into the ADP-bound state by nucleotide hydrolysis, and ADP release closes the cycle by returning the enzyme into the *apo* state. The transitions are stochastic in nature, and are associated to large-scale conformational movements of entire protein domains, resulting in random forces that play the role of a noise term that is additive to thermal fluctuations on the cantilever dynamics.

The model can be analytically solved (the resulting rates are presented in Tables S1 and S2 in File S1) and it fits well the data over the entire range of tested ATP concentrations (2 µM –0.2 mM –2 mM fits are shown in Figure 2). As is the case for several ATPase molecules, a single turnover of the ATPase cycle is completed in about 200 ms. Taking as an estimate an ATP encounter rate of about 10^5^–10^6^ s^−1^ M^−1^, the model predicts that the smaller of the hydrolysis and release rates is of the order 1–100 s^−1^. This also implies that ATP binding will be the slowest process at low ATP concentrations (2 µM) and possibly the fastest at the highest one (2 mM). Although it is not possible to extract each transition rate individually, the model predicts that the fastest between the hydrolysis and ADP release rates is approximately 50–100 s^−1^. This is in very good agreement with the rates present in literature obtained using conventional techniques [37], [38]. The present experiments can provide an easier and faster means to extract quantitative information about the cycle and some of its substeps (see File S1 for a more complete description of the model). The model that we have chosen here posits that the transitions are irreversible. A treatment with reversible transitions leads to the same functional form of the power-spectrum, although the presence of more cycle rates would make any identification more difficult. Moreover, the non-equilibrium nature of the phenomenon detected by the nanosensor ensures that one cycle direction, the one compatible with ATP hydrolysis, should prevail on the reverse direction, so that our modelling, although approximate, should capture the essential features of the system.

While Topo II is capable of maintaining a basal functionality, its activity benefits greatly from the presence of DNA molecules. Thus, we studied the fluctuations of the cantilever sensor when coated with Topo II-DNA complexes. Namely, we studied the fluctuations arising from the exposure to AMPPNP and ATP (Figure S9 in File S1). As expected, the results of such measurements demonstrate that the DNA indeed amplifies the Topo II cycle rate in our conditions by almost a factor 3. In these physiological conditions, we were able to perform also electrophoretic analyses that confirmed with this conventional technique, the functionality of the enzyme in our experimental conditions (Figure S10 in File S1).

Furthermore, by exploiting the high temporal resolution of the technique we were able to characterize the dynamics of the Topo II in presence and absence of DNA in the first seconds after the injection of the ATP. Figure S11 in File S1 compares the initial response of the Topo II-DNA complex (panel a) with the Topo II basal rate (panel b).

After investigating how two ligands (ATP and AMPPNP) can differently influence the dynamics of Topo II, we turned our attention to Aclarubicin (Aclar), an anticancer drug that specifically targets the function of the enzyme [21]. Two mechanisms of action have been proposed: either Aclar acts on the DNA duplex inhibiting its relaxation when interacting with Topo II [39], or it directly binds to the enzyme freezing its conformation [40], [41]. We have been able to study directly this phenomenon and to elucidate, for the first time, the effect of the drug.

**Figure 3:**
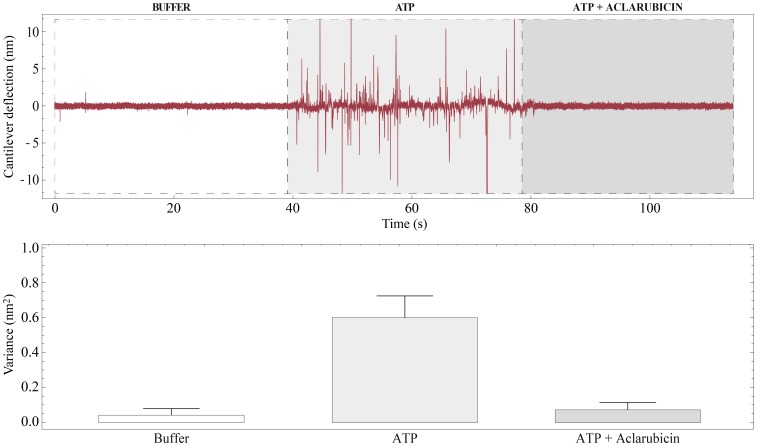
The Aclarubicin experiment. The cantilever deflections as different media were flowed through the analysis chamber: buffer, an ATP-enriched solution (20 µM), and finally a solution containing both ATP (20 µM) and Aclarubicin (100 µM). The variance values were calculated from five independent experiments.

Indeed, while ATP (20 µM) induced a strong deflection signal (compatible with the values obtained in previous experiments), the subsequent introduction of a solution containing both ATP and 100 µM of Aclar produced a fast decrease of the fluctuations whose amplitude returned, in seconds, to levels comparable to those obtained in the no-ATP cases (Figure 3). Since this effect was observed in absence of DNA, our experiments proved that Aclar affects directly the Topo II molecule inhibiting its conformational changes.

It is noteworthy to say that most of the previous experiments with Topo II in absence of DNA using bulk techniques have been difficult to interpret because of the low ATPase activity under those conditions [42]. Moreover, these experiments average the measurements over active and inactive proteins so the rates determined in ensemble measurements can be almost 1 order of magnitude smaller than the ones measured in single-molecule assays. New techniques and more precise measurements have allowed highlighting how, in the absence of DNA, there is a basal turnover rate whose intensity is not negligible when compared to the DNA-bound cases (see File S1 for a more in-depth discussion on this point) [36].

## Discussion and Conclusions

These results indicate that cantilever fluctuations are the result of the combination of energy dissipation (ATP hydrolysis) associated to conformational changes in the molecule. On one side, this is shown in the apyrase experiments; this enzyme binds and hydrolyses ATP with a very high rate but is so small that its folding produces no detectable fluctuation in the cantilever. On the other side, the results involving Topo II and Topo II-DNA complexes with AMPPNP indicate that if the Topo II undergoes a conformational change without energy consumption, this cannot be detected.

Furthermore, some similar experiments are present in literature. As an example, Schneider and coworkers used an AFM cantilever coated with myosin to probe the ATP present or expelled by a cellular membrane [43]. Also Radmacher and coworkers employed a cantilever to measure the hydrolysis-induced conformational changes of a single molecule [24]. These experiments demonstrate the potentiality of a nanomechanical sensor to characterize ATP hydrolysis by Topo II. Yet, since these pioneering experiments were carried out using an AFM in a conventional modality, several automatic feedback systems were activated to ensure that the tip-sample distance was maintained constant. This could introduce fluctuations or noise that might be mistakenly attributed to biological activity.

The method described in this work is similar to these cited systems and yet remarkably different: the cantilever is not placed in any vicinity to a surface and all the feedback loops are deactivated. We collect the fluctuations of the cantilever during the biological activity and we compare them to those produced by the environmental noise, to extract the contribution of the biological activity. This offers a new and original means to explore protein conformational changes and the dynamical effects of protein-ligand interactions with unparalleled sensitivity and temporal resolution. The use of parallel sensors (such as array of cantilevers) will open the door to a higher reliability of the measured effects, allowing a better control over possible unwanted side effects. Furthermore, it will increase the throughput and enable probing different experimental conditions at the same time. Finally, its intrinsic simplicity and versatility make this diagnostic tool a promising candidate to become a tabletop laboratory and clinical device.

From a broader perspective, the setup described here is a new addition to the growing list of “active matter” systems, where non-equilibrium, energy consuming, fluctuations are considered besides the more usual thermal, equilibrium ones. New properties can emerge, because of the breakdown of detailed balance and equilibrium fluctuation-dissipation theorems. A fruitful cross-fertilization of the present research and of the results in non-equilibrium statistical physics can be expected in the future.

## Supporting Information

File S1 Materials and methods, in depth description of the Topo II cycle, additional control experiments and the theoretical model. Additional figures, mainly regarding the characterization of the cantilever and the additional experiments.
(DOCX)
