# Supplementary material for: Real-Time Monitoring of Protein Conformational Changes Using a Nano-Mechanical Sensor
Source: PLoS One. 2014 Jul 31;9(7):e103674. doi: 10.1371/journal.pone.0103674 (PMC4117498; doi:10.1371/journal.pone.0103674)
Supplement: File S1 — Materials and methods, in depth description of the Topo II cycle, additional control experiments and the theoretical model. Additional figures, mainly regarding the characterization of the cantilever and the additional experiments. (DOCX) [file pone.0103674.s001.docx]

Real-time monitoring of protein conformational changes using a nano-mechanical sensor.

*Livan Alonso-Sarduy, Paolo De Los Rios, Fabrizio Benedetti, Dusan Vobornik, Giovanni Dietler, Sandor Kasas, Giovanni Longo.*

**Supplementary materials**

**Characterization of the cantilever**

The resonant frequency of the cantilevers was approximately 2 - 3 kHz in liquid. To verify that the measured fluctuation was positively caused by the interaction between the Topo II and the ATP, we compared the resonance frequencies and the deflection data of the cantilevers functionalized with APTES or with APTES and Topo II when immersed in the working buffer. The resonance frequencies of the cantilever were: bare cantilever: 1.99 ± 0.02 kHz; APTES-functionalized cantilever: 1.97 ± 0.03 kHz; and Topo II-functionalized cantilever: 1.93 ± 0.04 kHz. There was a small shift in the frequency when tips were


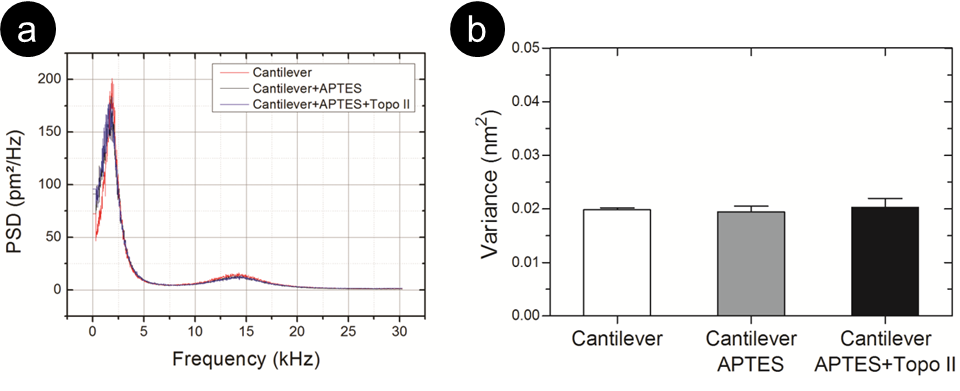


**Figure S1.** **Characterization of the functionalization of the cantilever.**

Panel a: Power Spectral Density data of the deflection signal for different functionalized cantilevers collected at the resonant frequency: bare cantilever, APTES-functionalized cantilever and Topo II-functionalized cantilever. All the PSDs were collected in PBS.

Panel b: Variance of the cantilever deflection data corresponding to three functionalized cantilevers. The Topo II concentration used to functionalize the cantilever was 107.7 nM. The variance data was collected from three independent experiments.


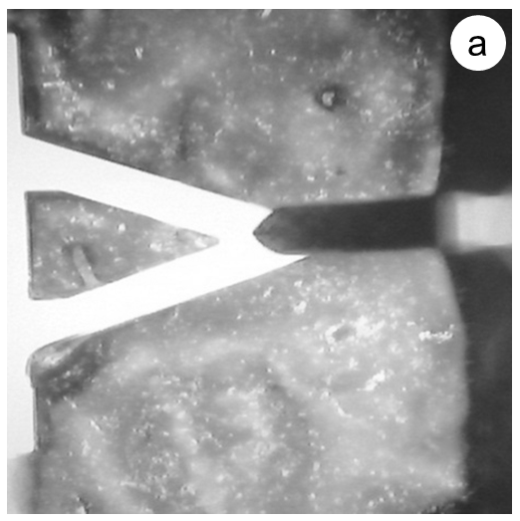

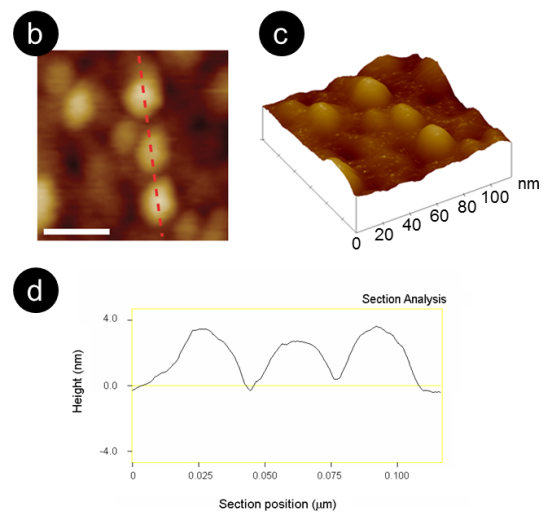


**Figure S2.** **High-resolution AFM characterization of the Topo II coverage of the cantilever surface.**

Panel a: Optical image of a cantilever collected during the AFM characterization.

Panel b:  A typical 100 x 100 nm topographic image of Topo II molecules deposited onto an APTES-functionalized cantilever (the scale-bar represents 40 nm). The Topo II concentration used to coat the cantilever was, in this case, 107.6 nM.

Panel c: 3D view of Topo II molecules.

Panel d: Cross-section profile collected along the dashed line shown in panel b.

functionalized with APTES and Topo II (**Figure S1a**); however, no differences were seen in the variance of the deflection data when Topo II was adsorbed onto the cantilever in absence of ATP (**Figure S1b**).

Since the amplitude of the deflection has a direct correlation to the number of Topo II molecules on the cantilever, we characterized the surface coverage prior to performing the experiments. For this purpose, as shown in **Figure S2a**, AFM was used to image the surface of the Topo‑functionalized cantilever and to estimate the surface occupancy of the molecules. Several high-resolution topography images were collected, evidencing a uniform coating of the sensor’s surface (**Figures S2b and c**). The mean height of these structures, obtained from these images, was 3.9 ± 0.5 nm (as indicated by the typical cross-section shown in **Figure S2d**) and is consistent with the dimensions of single Topo II molecules adhering to the cantilever. As illustrated in **Figures S3a and c**, both sides presented uniform and similar levels of occupancy of Topo II molecules. For a 107.6 nM Topo II concentration of the initial solution used to functionalize the cantilever, the results obtained were 685 ± 42 molecules/μm^2^ on the top side of the cantilever and 657 ± 39 molecules/μm^2^ on the bottom side. AFM images were analysed using ImageJ to determine the protein occupancy on the surface of the cantilevers (**Figures S3b and d**).


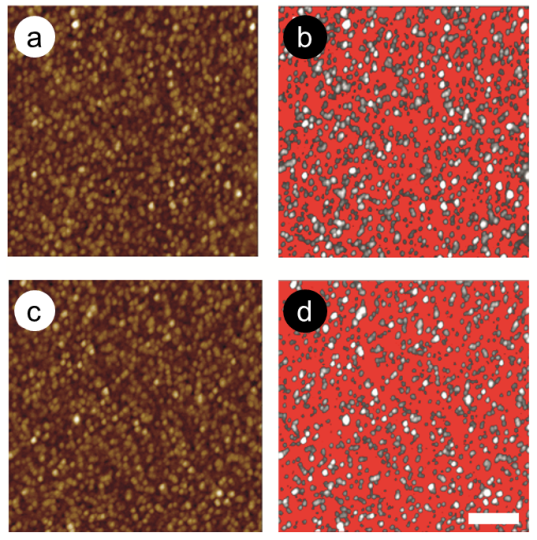


**Figure S3. Statistical analysis of the number of Topo II molecules.**

Panel a: Representative 1 x 1 μm AFM image of human Topo II molecules (107.6 nM) deposited on the top (gold coated) side of a cantilever.

Panel b: Particle analysis using ImageJ to identify the level of occupancy of Topo II performed on the image shown in Panel a.

Panels c and d: AFM characterization and image analysis performed on the bottom (non-coated) side of the cantilever. The scale-bar is common to all the images and represents 200 nm.

**DNA relaxation and gel electrophoresis**

DNA relaxation assays were performed as described by McClendon *et al*. [[44](#_ENREF_1)]. The samples were electrophoresed in 1% agarose containing 100 mM Tris-borate (pH 8.3) and 2 mM EDTA. The gels were stained with GelRed (Biotium) following the supplier’s instructions. The DNA bands were visualized with ultraviolet light using the Kodak® 1D Image Analysis software and quantified using ImageJ [[45](#_ENREF_2)].

**Additional information regarding the Topo II conformational changes**

Topo II undergoes cycles of ATP binding, ATP hydrolysis and ADP release, that produce conformational transitions promoting the sectioning of a duplex strand while passing a second one through the break and that, at the end of each cycle, religate the first DNA duplex strand[[46-49](#_ENREF_3)]. The ATPase region responsible for the DNA interaction contains GHKL domains, which are known to dimerize in response to ATP binding[[50](#_ENREF_7),[51](#_ENREF_8)]. This closes the protein clamp, trapping the DNA segment inside the N-gate of the enzyme and facilitating its exit through the DNA-gate[[52-54](#_ENREF_9)]. Structural studies have shown that nucleotide binding can induce a rotation of about 14º between the GHKL and the transducer domains in Topo IIA and that this movement is coupled with the positioning of the catalytic lysine into the ATPase site [[55](#_ENREF_12),[56](#_ENREF_13)]. The ATPase reaction regulates the steps in the reaction cycle in a way that is otherwise thermodynamically unfavourable, even though it is known that, at equilibrium, a fraction of these domains are closed.

Kinetic analyses have demonstrated that the entire cycle of Topo II does not require the presence of the DNA molecules which simply amplify the cycle rate. Even in absence of DNA there is a basal turnover rate, whose intensity is less than 1 order of magnitude smaller than that measured in the DNA-bound cases[[57-60](#_ENREF_14)]. For example, Campbell shows that the basal activity with increasing ATP concentration reaches as high as one sixth of the DNA-bound topoisomerase activity[[57](#_ENREF_14)], while Hammonds measures a 8x stimulation of the basal ATPase rate when adding DNA[[61](#_ENREF_18)] and finally Schmidt evidences a 5.5x stimulation of the basal level when DNA is bound[[59](#_ENREF_16)]. The difficulty to determine this basal level could arise from the techniques used to study the ATPase turnover of the Topo II: bulk experiments yield enzymatic activity values that are averaged over active and inactive proteins. Such ensemble measurements can show rates almost 1 order of magnitude smaller than the ones measured in single-molecule assays[[62](#_ENREF_19),[63](#_ENREF_20)]. Instead, the methodology described in the present work measures the rate of only the bound and active enzymes and allowed detecting the activity of Topo II both in presence and in absence of DNA. In fact, this DNA-induced stimulation is strongly dependent on the environmental conditions; Harkins shows that the ATPase stimulation depends on the concentration of potassium acetate (KOAc) in the working buffer, and that, in the case of no KOAc, there is no difference between the basal and the stimulated rate[[58](#_ENREF_15)]. In our conditions, we have compared the fluctuation amplitude induced by the Topo II (**Figure 1** in the 20 μM case) and the Topo II-DNA complexes (**Figure S9**) and we were able to estimate a DNA-induced amplification of less than a factor three.

**Theoretical estimation of the energies involved**

If we consider the cantilever as a spring, with a constant of $\text{k=0.06}\frac{\text{N}}{\text{m}}$, the energy needed to produce a 1 nm average deflection of this spring is:

$E=\frac{1}{2}k\cdot\left\langle x\right\rangle^{2}=3\cdot10^{-20}$J

From the energy point of view, a single ATP hydrolysis event produces approximately 5*10^-20^ J per molecule[[64](#_ENREF_21)] (other works and textbooks claim the energy produced is ~30 kJ per mole, which computes similar energies per molecule[[65](#_ENREF_22)]).

This indicates and confirms that, energetically, even few Topo II molecules hydrolyzing each two ATPs would produce enough energy to deflect the cantilever as we have measured in our experiments. Since the functionalization procedure cannot ensure that all the Topo II molecules are concentrated on the apical area of the cantilever, (and, in fact, **Figure S2 and S3** have shown that they are uniformly distributed all over the cantilever), this approximation can be considered valid only as an illustration of the energy needed to cause the fluctuations of the cantilever. If the force is applied on the apex of the cantilever, it will have maximum efficiency; while at the base of the cantilever its effect will be less than 10%[[66](#_ENREF_23)]. In any case, this rough calculation demonstrates that a concurrent hydrolysis-induced conformational change of few molecules oriented orthogonally to the cantilever surface will be sufficient to produce an average fluctuation of the cantilever of the measured amplitude. Naturally, since each Topo II molecule performs a folding event independently and the force it produces is oriented randomly, such concurrent event is extremely rare. Thus, to achieve this effect in timescales compatible with our experimental analyses we need almost 10 million molecules attached to the lever.

**Additional experiments**

To determine the cause of the sensor’s deflections we investigated the steps that ultimately cause the Topo II conformational change. We performed several control experiments to estimate the effect of the exposure of bare cantilevers to the different media we used for our investigations. We also focused on the ATP hydrolysis, studying the effect of a non-hydrolysable ATP-analogue and of Caged-ATP on Topo II and the effect of an ATPase molecule, which does not undergo conformational changes. Finally, we compared the basal and the DNA-amplified rates by studying the fluctuations induced by the Topo II-DNA complexes.

**Experiments involving a low concentration of Topo II exposed to ATP**

We studied the effect of the concentration of Topo II on the cantilever on the resulting amplitude of the fluctuations. We prepared the cantilever with a low concentration of Topo II (53.8 nM, half of the concentration used in the experiments described in **Figure 1b and c**) and we introduced it in the analysis chamber. We collected its fluctuations as a function of

**Figure S4. The experiment involving a low Topo II concentration (53.8 nM) exposed to ATP.** The cantilever was coated with a low concentration (53.8 nM) of Topo II. The deflections and corresponding variance values are shown as a function of ATP concentration. Different media were flowed through the analysis chamber: the working buffer (with no ATP), ATP-enriched buffer, containing in order 0.2 μM, 2.0 μM, 0.02 mM, 0.2 mM and 2.0 mM ATP, and then again the working buffer.

time while several solutions containing increasing concentrations of ATP were consecutively flowed through the chamber at a rate of 4 µl/s. The first and last periods corresponded to the buffer without ATP. The results of these experiments are shown in **Figure S4**: increasing the concentration of ATP the fluctuations increased and returned to low levels when the ATP was flushed out of the chamber. The absolute values of these fluctuations were reduced compared to the high Topo II experiments, but the overall dependence on the ATP concentration is maintained.

**Experiments involving Topo II exposed to Caged-ATP**

We used a photo-activating ATP to confirm that the cantilever fluctuations measured using our system were, indeed, caused by a hydrolysis-driven reaction between the ATP and the Topo II. We prepared the cantilever with a high concentration of Topo II and we exposed it first to the working buffer and then to a solution containing 20 μM of Caged-ATP. At this point the fluctuations of the cantilever indicated that the system was only barely influenced by the injection of the inactivated molecules (the small increase in variance is probably caused by a small portion of uncaged molecules in the solution). Upon exposure to UV light, the fluctuations increase rapidly and remain high even after the UV light is turned off (**Figure S5**). The physical and optical properties of the medium before and after the exposure to the UV light are identical, and this rules out any interpretation of the origin of the fluctuations that does not involve the Topo II hydrolysis of the ATP.

**Figure S5. The experiment involving Topo II exposed to Caged-ATP.**The Topo II coated cantilever deflections and corresponding variance as a function of the presence and activation of Caged-ATP. The analysis chamber was first flowed with the working buffer (with no Caged-ATP) and subsequently with the Caged-ATP-enriched solution (20 μM). When in this latter state, a UV lamp was used to activate the Caged-ATP and start the hydrolization cycles. The slight increase in fluctuations when the unactivated Caged-ATP was introduced is probably caused by a small percentage of activated molecules.

**Experiments involving Topo II exposed to AMPPNP**

To determine the origin of the fluctuations we studied a system that is known to cause conformational change in Topo II, but with no associated release of energy. We chose a non-hydrolysable ATP-analogue (AMPPNP). Given its identical size and very similar structure, a solution containing a small concentration of AMPPNP has very similar optical[[67](#_ENREF_24)] and physical properties (especially at such low concentrations) as one containing the same concentration of ATP.[[68](#_ENREF_25)] Similarly, to the ATP, the AMPPNP binding induces dimerization of the ATPase regions, triggering a conformational change of the homodimeric enzyme, but trapping the Topo II in a closed clamp conformation[[52](#_ENREF_9)]. In the presence of AMPPNP most of the enzyme is converted to a state in which DNA binding and release are extremely slow but which allows DNA cleavage[[60](#_ENREF_17)]. Such conformational change is not associated with hydrolysis; therefore there is no net energy production in this cycle. The results of these experiments are shown in **Figure S6**: increasing the concentration of AMPPNP does not induce a measurable variation of the fluctuation amplitude and of the associated variance. In conclusion, this is the main difference between ATP-induced cycles and AMPPNP-induced cycles: in one case, the energy produced is more than enough to induce a deflection of the cantilever of several nanometers, while, in the other, no energy is released and no deflections can be seen (apart from the thermally-induced fluctuations).

**Figure S6. The experiment involving Topo II exposed to AMPPNP.** The Topo II cantilever deflections and corresponding variance as a function of AMPPNP concentration. Different media were flowed through the analysis chamber: the working buffer (with no AMPPNP), AMPPNP-enriched buffer, containing in order 0.2 μM, 2.0 μM, 0.02 mM, 0.2 mM and 2.0 mM ATP, and then again the working buffer. The corresponding variance values were obtained from three independent experiments. The cantilever was coated using 107.7 nM Topo II.

**Experiments involving Apyrase**

A second series of experiments focused on the determination of the role of the hydrolysis in the arising of the fluctuations involved apyrase, a small molecule (~47 kD vs ~180 kD of the Topo II) that is a very effective ATPase, hydrolyzing ATP in ADP with a very high efficiency[[69](#_ENREF_26)]. The cantilever was coated with apyrase and inserted in the analysis chamber. As shown in **Figure S7** the exposure to a buffer containing 2 mM ATP induced no increase of the variance

**Figure S7. The experiment involving apyrase exposed to ATP.** The cantilever was coated with apyrase. The deflections and corresponding variance values are shown as a function of the ATP presence. The analysis chamber was first flowed with the working buffer (with no ATP) then with ATP-enriched buffer (20 μM) and again with the working buffer.

level of the fluctuations. The only deflections of the cantilever were caused by the thermal fluctuations and appeared identical before and after the injection of ATP. Thus, ATP hydrolysis *per se*, without large conformational changes, is unable to induce cantilever fluctuations.

**Figure S8. The experiments involving the bare cantilever.** The different experiments were performed using cantilevers functionalized with APTES but with no Topo II attached to them. The cantilevers in different experiments were exposed to ATP (Panel a), AMPPNP (Panel b) and Aclarubicin (Panel c) and the resulting fluctuations were measured.

**Control experiments**

To characterize the effect on the cantilever fluctuations of the different physical or optical properties of the media used in our experiments we performed several experiments involving bare and functionalized cantilevers in absence of Topo II. We exposed these control cantilevers to ATP, AMPPNP and Aclarubicin and measured the resulting fluctuations (**Figure S8**). In all these control experiments the only measured effect was the introduction of static deflections of the cantilever that did not in any way influence the outcome of the measurements. Since the variance and the PSD of the fluctuations depend only on the amplitude of the cantilever around its average value and not on the average value itself, such slow changes over time do not modify these parameters.

**The experiments involving the Topo II-DNA complexes**

To study the effect of the presence of DNA in the conformational change rate of Topo II, we immobilized the Topo II-DNA complex on the sensor and then exposed it to AMPPNP (20 μM) and then ATP (20 μM).

These measurements show that, in presence of AMPPNP, the fluctuations had amplitude comparable to those measured when only the working buffer was present. On the other hand, when the buffer containing ATP was injected in the analysis chamber the fluctuations increased and their absolute value was almost three times higher than the basal rate measurement (compare **Figure 1** and **Figure S9,** at the same 20 μM ATP concentration).

**Figure S9. The experiment involving the Topo II-DNA exposed to AMPPNP and ATP.** The cantilever was coated with a Topo II-DNA complex and exposed to the working buffer, buffer enriched with 20 μM AMPPNP and finally buffer enriched with 20 μM ATP. The deflections and corresponding variance values are shown as a function of the medium.


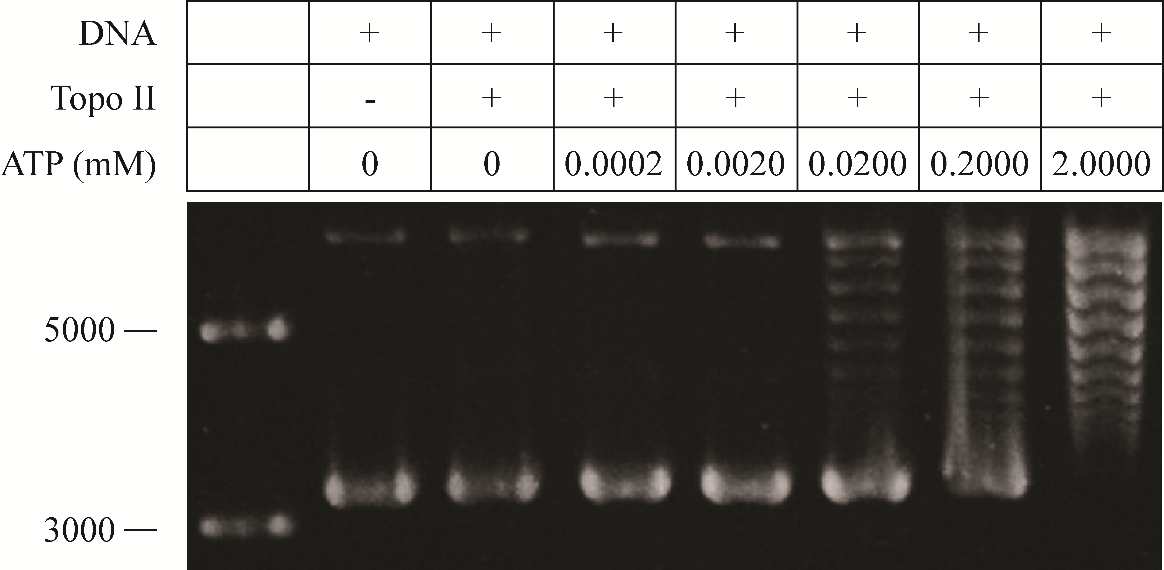


**Figure S10. Electrophoretic analysis.** Electrophoresis of negatively supercoiled pBR322 plasmids after Topo II-induced relaxation in a medium containing different concentrations of ATP. DNA molecular weight markers are indicated on the left of the gel.

To study with conventional techniques the functionality of the Topo II enzymes we performed several control experiments using other complementary techniques. For instance, some of us have used AFM to image, in liquid, the functionality of the Topo II molecules after immobilization on a surface [[70](#_ENREF_27)]. These in these time-lapse images we have shown the different conformations of the Topo II molecules without the presence of DNA and the effect of Topo II on DNA molecules. In other control experiments, we performed electrophoresis on negatively supercoiled pBR322 plasmids in presence of Topo II at different ATP concentrations. The results, presented in **Figure S10**, show that the increase of ATP induces an augmentation of the Topo II activity.

As described above, after the injection of a new liquid medium in the analysis chamber we have always allowed a stabilization period (usually 1 minute). While this produces a stable measurement of the fluctuations, it also causes the loss of the information regarding the first cycles of the Topo II molecules. Since the high temporal resolution of this technique can deliver a characterization of the phenomena under investigation that is unachievable using other techniques, we performed some experiments to investigate these first instants of the interaction. We chose to measure the fluctuations while maintaining a very slow and constant injection rate (0.1 μl/second), in order to reduce to a minimal constant value the pump-induced noise throughout the entire measurement. At this rate the chamber was first of all filled with the working buffer. Next, the ATP-enriched buffer was injected. In this way we measured the fluctuations induced by the Topo II when the first ATP-enriched

**Figure S11. The comparison between the first cycles of the Topo II-DNA and of the Topo II.**

Panel a: The first seconds of the interaction between the Topo II-DNA complexes and the ATP. The injection system was activated at a very slow rate while the measurement was underway. First the analysis chamber and the tubes were filled with the working buffer and next the ATP-enriched buffer was pumped. The resulting diagram records the immediate response of the complex to the exposure to ATP. The dashed horizontal line represents the constant average level measured after this transient.

Panel b: The very same experiment performed using sensors covered with Topo II without any DNA.

medium reached the cantilever. These fluctuations detail the effect of the first Topo II conformational changes and we compared these time-resolved diagrams in the case of Topo II alone and of Topo II-DNA complexes. The measurements in the DNA case highlight a well-defined activity spike that lasts for few tens of seconds and then stabilizes to a smaller stable value (**Figure S11 panel a**). This is probably a reflection of the presence of DNA on the cantilever before the injection of the ATP: at first all the Topo II molecules are loaded with DNA and the resulting first cycle will be shorter (the DNA is already loaded in the Topo II) and with higher amplitude, since all the cycles will be somewhat synchronized. After this, some of the DNA will be displaced and in some cases detached from the cantilever surface and thus will be lost to the measurable reaction. This means that not all the Topo II will have access to the DNA. The resulting fluctuation amplitude will stabilize to a constant and smaller rate, which will average to almost three times the amplitude of the basal rate. On the other hand, in the no-DNA case (**Figure S11 panel b**) the first cycles will be identical to the subsequent ones and this will result in the more uniform basal rate.

**Model**

**Dynamical model of the cantilever.**

The cantilever can be modeled as a spring of mass *m*, constant *k* and subject to a damping friction *γ* with the surrounding fluid. Calling *z* the vertical displacement of the cantilever, the governing equation is

where *η *is a Gaussian variable of zero mean and unitary variance, with its prefactor fixed by the fluctuation-dissipation theorem. The statistical properties of *η *are then defined by its statistical autocorrelation function, typically taken as uncorrelated white noise:

$$\left\langle\eta\left(t'\right)\eta\left(t\right)\right\rangle=\delta\left(t-t'\right)$$

The solution of this equation is more easily obtained in Fourier space to give

$$\tilde{z}\left(\omega_{k}\right)=\frac{\sqrt{2\gamma k_{B}T}\tilde{\eta}\left(\omega_{k}\right)}{-m\omega_{k}^{2}+i\gamma\omega_{k}+k}$$

with $\omega_{k}=2\pi k/t_{TOT}$ ($t_{TOT}$ being the duration of times series).

The average power spectrum of *z* is

$$PSD\left[z\right]\left(\omega_{k}\right)=\left\langle\tilde{z}\left(\omega_{k}\right)\cdot\tilde{z}^{*}\left(\omega_{k}\right)\right\rangle=\frac{2\gamma k_{B}T}{m\left[\left(\omega_{k}^{2}-\frac{k}{m}\right)^{2}+\left(\frac{\gamma}{m}\right)^{2}\omega_{k}^{2}\right]}$$

When a further random force, uncorrelated from the thermal one, is acting on the cantilever, the new dynamical equation becomes

where *F(t)* is, in principle, not subject to the fluctuation-dissipation theorem, so that its statistical and spectral properties are, for the moment, unspecified.

The average power spectrum of *z* is then

$$PSD\left[z\right]\left(\omega_{k}\right)=\left\langle\tilde{z}\left(\omega_{k}\right)\cdot\tilde{z}^{*}\left(\omega_{k}\right)\right\rangle=\frac{2\gamma k_{B}T}{m\left[\left(\omega_{k}^{2}-\frac{k}{m}\right)^{2}+\left(\frac{\gamma}{m}\right)^{2}\cdot\omega_{k}^{2}\right]}\left[1+\frac{1}{2\gamma k_{B}T}PSD\left[F\right]\left(\omega_{k}\right)\right]$$

The next section describes a model that we use to predict the structure of *F(t)*.

**Model of the protein-related random force**

We assume that proteins on the cantilever can access three states, namely apo (no bound nucleotide), ATP (bound ATP) and ADP (bound ADP). The transitions take place at rates *k_h_* (hydrolysis: ATP→ADP), *k_off_* (ADP release: ADP→*apo*) and *k_on_*=*k_enc_*[ATP] (ATP binding: *apo*→ATP) where *k_enc_* is the encounter rate between a protein and one ATP molecule, as depicted in **Figure S12**. For simplicity, we assume that the three transitions are irreversible. This is for clearly correct for ATP hydrolysis, which entails the breakage of covalent bonds, and for ADP release, as the ADP concentration remains very small in the course of the experiment making ADP rebinding extremely unlikely; as well, we expect that the high ATP concentrations make ATP binding overwhelming with respect to ATP release. At any rate, our results are independent on these assumptions. The number of molecules in each of the three states at time *t* is *N*_apo_(*t*), *N*_ATP_(*t*), *N*_ADP_(*t*). Customarily, the evolution of the populations obeys the rate equations


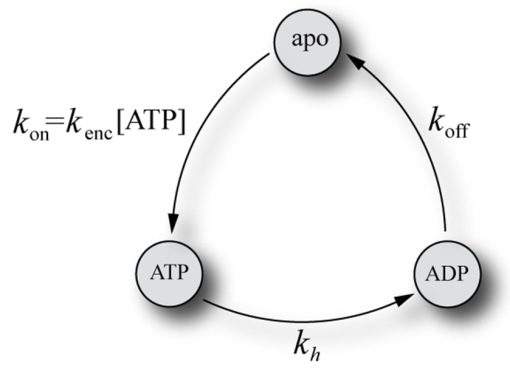


**Figure S12. The model: a schematic representation of the topoisomerase II cycle.** The protein can populate three states depending on its nucleotide state. The three transitions are considered, for simplicity, to be irreversible. The ATP binding reaction explicitly depends on the ATP concentration.

It is worth stressing that the sum of the three equations yields

because the total number *N* of proteins on the cantilever, that are susceptible to enter the cycle, is conserved. Therefore, only two of these equations are linearly independent and needed for the solution.

The stationary solution of the rate equations is then

$$\bar{N}_{ATP}=\frac{k_{on}k_{off}}{k_{on}k_{off}+k_{on}k_{h}+k_{h}k_{off}}N$$

$$\bar{N}_{ADP}=\frac{k_{on}k_{h}}{k_{on}k_{off}+k_{on}k_{h}+k_{h}k_{off}}N$$

$$\bar{N}_{apo}=\frac{k_{h}k_{off}}{k_{on}k_{off}+k_{on}k_{h}+k_{h}k_{off}}N$$

At steady state, the values $\bar{N}_{ATP},\bar{N}_{ADP} \text{and} \bar{N}_{apo}$ are constant, giving only a zero frequency contribution to the power spectrum.

The correct description of the process, that instead has a rich time- and frequency-domain behavior, is based on a more fundamental interpretation of the rates. Taking the ATP→ADP transition as an example, the rate *k_h_* is related to the probability *k_h_*Δ*t* that a single protein undergoes the transition in a small time-interval Δ*t*. If we assume that each protein does (or does not) undergo the transition independently on the others, the probability of having *n* proteins hydrolyzing ATP between times *t* and *t*+Δ*t* is

$$P\left(n,t,\Delta t\right)=\left(\begin{matrix} N_{ATP}\left(t\right) \\ n \end{matrix}\right)\left(k_{h}\Delta t\right)^{n}\left(1-k_{h}\Delta t\right)^{N_{ATP}\left(t\right)-n}$$

and the average value is $n\left(t+\Delta t,t\right)=k_{h}\Delta t\cdot N_{ATP}\left(t\right)$, which leads indeed to the usual rate equations written above, highlighting that they describe the evolution of the average number of proteins in each state, rather than the exact instantaneous numbers of proteins. In fact, at any time the precise number of proteins undergoing the transition is not exactly identical to the average. It will be equal instead to a random variable from a distribution centered on $n\left(t+\Delta t,t\right)$ and with variance

$\sigma_{h}^{2}\left(t+\Delta t,t\right)=N_{ATP}\left(t\right)k_{h}\Delta t\left(1-k_{h}\Delta t\right)$.

Analogous results hold for the other transitions. With this in mind, we can now rewrite the rate equations in the form

.

where the *noise* terms $\xi_{i}\left(t\right)$ (*i*=ATPADP or apo) are random variables of zero mean and unitary variance, and time correlations

$\left\langle\xi_{i}\left(t'\right)\xi_{j}\left(t\right)\right\rangle=\delta\left(t'-t\right)\delta_{ij}$.

These new *stochastic* rate equations, governing the evolution of the populations of the three states, are **stochastic equations with multiplicative noises**. Typically, these sets of equations cannot be analytically solved because the multiplicative part of the noise is a square root of the dynamical variables of interest. To address this issue we apply a series of simplifying assumptions. First, at any instant the number of molecules in a given state is a fluctuation around the stationary state, namely $N_{i}\left(t\right)=\bar{N}_{i}+\delta N_{i}\left(t\right)$ (*i*=ATP, ADP or *apo*). The stochastic rate equations become

If we then assume that $\delta N_{i}\left(t\right)/\bar{N}_{i}\ll1$ then the equations finally become

In order to simplify the notation we define the following quantities

$$\delta n_{i}\left(t\right)=\delta N_{i}\left(t\right)/\sqrt{N}$$

$$\begin{matrix} \sigma^{2}=k_{h}k_{on}k_{off}/\left(k_{on}k_{off}+k_{h}k_{on}+k_{h}k_{off}\right) \\ \sigma_{ATP}^{2}=k_{h}\left(k_{on}k_{off}+k_{on}k_{h}+k_{h}k_{off}\right)/\left(4k_{on}k_{off}\right) \\ \sigma_{ADP}^{2}=k_{off}\left(k_{on}k_{off}+k_{on}k_{h}+k_{h}k_{off}\right)/\left(4k_{on}k_{h}\right) \\ \sigma_{apo}^{2}=k_{on}\left(k_{on}k_{off}+k_{on}k_{h}+k_{h}k_{off}\right)/\left(4k_{h}k_{off}\right) \end{matrix}$$

so that the equations become at last

As mentioned above, these are three coupled **stochastic equations with correlated additive and multiplicative noises**. Although they are simpler than the original set of equations with square root multiplicative noise, they are still extremely cumbersome. We thus proceed to a further simplification: since we expect σ to be of the same order of *σ*_ATP_, *σ*_ADP_ and *σ*_apo_ and *δn*_i_ ~ O(1) (*i=*ATP, ADP or apo) at most, we can expect the multiplicative noise terms to be negligible with respect to the additive ones if *N* ≫ 1.

The equations thus reduce to

.

Using the conservation of the number of proteins we have $\delta n_{ATP}+ \delta n_{ADP}+\delta n_{apo}=0$ and the number of equations can be reduced:

Interestingly, although the number of proteins in the *apo* state is explicitly absent, the noise term it contributes to the fluctuations is still present.

In order to solve this problem we Fourier transform the system of equations. The stochastic rate equations become

$$\left\{\begin{matrix} \left[i\omega_{k}+\left(k_{h}+k_{on}\right)\right]\delta\tilde{n}_{ATP}+k_{on}\delta\tilde{n}_{ADP}=\sigma\left(\tilde{\xi}_{apo}-\tilde{\xi}_{ATP}\right) \\ -k_{h}\delta\tilde{n}_{ATP}+\left[i\omega_{k}+k_{off}\right]\delta\tilde{n}_{ADP}=\sigma\left(\tilde{\xi}_{ATP}-\tilde{\xi}_{ADP}\right) \end{matrix}\right.$$

The solution to this equation is

$$\left\{\begin{matrix} \delta\tilde{n}_{ATP}\left(\omega_{k}\right)=-\sigma\frac{\left(i\omega_{k}+k_{off}\right)\left(\tilde{\xi}_{apo}-\tilde{\xi}_{ATP}\right)-k_{on}\left(\tilde{\xi}_{ATP}-\tilde{\xi}_{ADP}\right)}{\left[\omega_{k}-i\left(k_{3}+\Delta\right)\right]\left[\omega_{k}-i\left(k_{3}-\Delta\right)\right]} \\ \delta\tilde{n}_{ADP}\left(\omega_{k}\right)=-\sigma\frac{k_{h}\left(\tilde{\xi}_{apo}-\tilde{\xi}_{ATP}\right)+\left(i\omega_{k}+k_{h}+k_{on}\right)\left(\tilde{\xi}_{ATP}-\tilde{\xi}_{ADP}\right)}{\left[\omega_{k}-i\left(k_{3}+\Delta\right)\right]\left[\omega_{k}-i\left(k_{3}-\Delta\right)\right]} \end{matrix}\right.$$

where we have set

$$k_{3}=\frac{k_{h}+k_{off}+k_{enc}\left[ATP\right]}{2}$$

$$\Delta=\frac{1}{2}\sqrt{k_{h}^{2}+k_{enc}^{2}\left[ATP\right]^{2}+k_{off}^{2}-2\left(k_{off}k_{h}+\left(k_{h}+k_{off}\right)k_{enc}\left[ATP\right]\right)}$$

and it is important to stress that Δ can be real as well as imaginary, depending on the values of the various rates. Of course, due to the conservation of the total number of proteins participating to the cycle,

$$\delta\tilde{n}_{apo}=-\delta\tilde{n}_{ATP}-\delta\tilde{n}_{ADP}$$

We want to estimate the force that can be generated by proteins during their transitions. Assuming that each transition is generating a f force *f_i_* (*i* = *h*, *off* or *on*), the force that is applied to the cantilever per unit time is

$$F\left(t\right)=\left[f_{h}k_{h}\delta n_{ATP}\left(t\right)+f_{off}k_{off}\delta n_{ADP}\left(t\right)+f_{on}k_{on}\delta n_{apo}\left(t\right)\right]\sqrt{N}$$

and, in Fourier space,

$$\tilde{F}\left(\omega_{k}\right)=\left[f_{h}k_{h}\delta\tilde{n}_{ATP}\left(\omega_{k}\right)+f_{off}k_{off}\delta\tilde{n}_{ADP}\left(\omega_{k}\right)+f_{on}k_{on}\delta\tilde{n}_{apo}\left(\omega_{k}\right)\right]\sqrt{N}$$

The power-spectrum of *F* is then

$$PSD\left[F\right]\left(\omega_{k}\right)=\frac{k_{h}k_{off}}{k_{h}+k_{off}}\frac{\left[ATP\right]}{K_{D}+\left[ATP\right]}\frac{A\omega_{k}^{2}+B}{\omega_{k}^{4}+a^{2}\omega_{k}^{2}+b^{4}}N$$

with

$$K_{D}=\frac{k_{h}k_{off}}{\left(k_{h}+k_{off}\right)k_{enc}}$$

$$\begin{matrix} A=\left(f_{h}k_{h}-f_{on}k_{enc}\left[ATP\right]\right)^{2}+\left(f_{h}k_{h}-f_{off}k_{off}\right)^{2}+\left(f_{off}k_{off}-f_{on}k_{enc}\left[ATP\right]\right)^{2} \\ B=\left\{k_{h}\left(f_{on}k_{enc}\left[ATP\right]-f_{off}k_{off}\right)+k_{enc}\left[ATP\right]\left(f_{h}k_{h}-f_{off}k_{off}\right)\right\}^{2} \\ +\left\{k_{enc}\left[ATP\right]\left(f_{off}k_{off}-f_{h}k_{h}\right)+k_{off}\left(f_{on}k_{enc}\left[ATP\right]-f_{h}k_{h}\right)\right\}^{2} \\ +\left\{k_{off}\left(f_{h}k_{h}-f_{on}k_{enc}\left[ATP\right]\right)+k_{h}\left(f_{off}k_{off}-f_{on}k_{enc}\left[ATP\right]\right)\right\}^{2} \end{matrix}$$

and

$$a^{2}=2\left(k_{3}^{2}+\Delta^{2}\right)$$

$$b^{4}=\left(k_{3}^{2}-\Delta^{2}\right)^{2}$$

where the sign of *a* depends on the sign and amplitude of Δ^2^.

Fitting the power spectra for different ATP concentrations (from 2 μM to 2mM) we can estimate the various parameters (as depicted in **Figure 2**). In particular the power spectrum amplitudes at *ω_k_*=0 should be a sigmoidal function of [ATP]. Indeed a sigmoid is a good fit, providing an estimate for *K_D_*≃8⋅10^-5^ M (± 3⋅10^-5^ M). Because the term *k_h_ k_off_ /*( *k_h_ + k_off_* ) is of the order of the smallest of the two rates, we can right away infer that the ratio between *k_enc_* and the smallest of the two other rates is 10^4^-10^5^. It is known that the rate of ATP hydrolysis can depend on the particular experimental conditions[[57](#_ENREF_14),[59](#_ENREF_16)].Yet, taking as an estimate an encounter rate of about 10^5^-10^6^ s^-1^ M^-1^ (as inferred by the nucleotide binding rates of other ATPase molecules, such as Hsp70[[71](#_ENREF_28)] and GroEL[[72](#_ENREF_29)]), the model predicts that the smaller of the other two rates is of the order 1-100 s^-1^. This also implies that ATP binding will be the slowest process at low ATP concentrations (2 μM) and possibly the fastest at the highest one (2 mM).

The values of A from the experiments are always several orders of magnitude smaller than the corresponding values of B, which again leads to at least one of the rates being of the order of 100 s^-1^.

To corroborate such findings we analyzed the values of *a* and *b* from the experiments (summarized in **Table S1**) and we can extract values for *k_3_* and Δ^2^ (**Table S2**)

Since *k_3_* is of the same order of the fastest rate, we infer, consistently with our previous conclusions, that the fastest rate is of the order of 100 s^-1^ even at low ATP concentrations (corresponding to a nucleotide binding rate at least one order of magnitude smaller). Thus, we can safely claim that either ATP hydrolysis or ADP release must match the 100 s^-1^ timescale.

The negative values of Δ^2^ complement such scenario by highlighting that there cannot be a single rate which dominates the others. Rather, at least two rates must be similar to allow for a compensation that leads to a negative value. Using again a nucleotide-Topo II encounter rate of 10^5^-10^6^ M^-1^ s^-1^, it turns out that the two comparable rates must be of the order of 100 s^-1^ (in particular using the two values of Δ^2^ for the largest nucleotide concentrations, where uncertainties seem to be the smallest).

Finally, it must be noted that, while the model describes remarkably well the overall phenomenon under investigation, some discrepancies can be identified, especially at the highest ATP concentration. Indeed, at 2 mM, the PSD appears to be steeper, following probably a ω^6^ power law. This indicates that at higher ATP values the model should be modified with additional steps in the protein cycle or by preserving the multiplicative noise component.

**Supplementary Tables and captions**

| ATP concentration (μM) | a (s^-1^) | b (s^-1^) |
| --- | --- | --- |
| 2 μM | 93 (8%) | 74 (3%) |
| 0.2 mM | 56 (8%) | 68 (2%) |
| 2 mM | 79 (9%) | 90 (2%) |

**Table S1. Results of the model.** Values of the variables a and b for the analyzed ATP concentration values. These results were obtained from the analysis of the experimental data using the model.

| ATP concentration (μM) | *k_3_* (s^-1^) | Δ^2^ (s^-2^) |
| --- | --- | --- |
| 2 μM | 70 (3%) | -575 (88%) |
| 0.2 mM | 55 (3%) | -1528 (15%) |
| 2 mM | 74 (4%) | -2489 (18%) |

**Table S2. Results of the model.** Values of the variables *k_3_* and Δ^2^ for the analyzed ATP concentration values. These results were obtained from the analysis of the experimental data using the model.

**Additional References**

44. McClendon AK, Rodriguez AC, Osheroff N (2005) Human topoisomerase IIalpha rapidly relaxes positively supercoiled DNA: implications for enzyme action ahead of replication forks. J Biol Chem 280: 39337-39345.

45. Schneider CA, Rasband WS, Eliceiri KW (2012) NIH Image to ImageJ: 25 years of image analysis. Nature Methods 9: 671-675.

46. Champoux JJ (2001) DNA topoisomerases: Structure, function, and mechanism. Annual Review of Biochemistry 70: 369-413.

47. Classen S, Olland S, Berger JM (2003) Structure of the topoisomerase II ATPase region and its mechanism of inhibition by the chemotherapeutic agent ICRF-187. Proceedings of the National Academy of Sciences of the United States of America 100: 10629-10634.

48. Schultz P, Olland S, Oudet P, Hancock R (1996) Structure and conformational changes of DNA topoisomerase II visualized by electron microscopy. Proceedings of the National Academy of Sciences of the United States of America 93: 5936-5940.

49. Berger JM, Gamblin SJ, Harrison SC, Wang JC (1996) Structure and mechanism of DNA topoisomerase II. Nature 379: 225-232.

50. Prodromou C, Panaretou B, Chohan S, Siligardi G, O'Brien R, et al. (2000) The ATPase cycle of Hsp90 drives a molecular 'clamp' via transient dimerization of the N-terminal domains. Embo Journal 19: 4383-4392.

51. Ban C, Junop M, Yang W (1999) Transformation of MutL by ATP binding and hydrolysis: A switch in DNA mismatch repair. Cell 97: 85-97.

52. Roca J, Wang JC (1992) The capture of a DNA double helix by an ATP-dependent protein clamp - A key step in DNA stransport by type-II DNA topoisomerases. Cell 71: 833-840.

53. Bates AD, Berger JM, Maxwell A (2011) The ancestral role of ATP hydrolysis in type II topoisomerases: prevention of DNA double-strand breaks. Nucleic Acids Research 39: 6327-6339.

54. Bates AD, Maxwell A (2010) The role of ATP in the reactions of type II DNA topoisomerases. Biochem Soc Trans 38: 438-442.

55. Corbett KD, Berger JM (2004) Structure, molecular mechanisms, and evolutionary relationships in DNA topoisomerases. Annual Review of Biophysics and Biomolecular Structure 33: 95-118.

56. Wigley DB, Davies GJ, Dodson EJ, Maxwell A, Dodson G (1991) Crystal-Structure Of An N-Terminal Fragment Of The DNA Gyrase B-Protein. Nature 351: 624-629.

57. Campbell S, Maxwell A (2002) The ATP-operated clamp of human DNA topoisomerase II alpha: Hyperstimulation of ATPase by "piggy-back" binding. Journal of Molecular Biology 320: 171-188.

58. Harkins TT, Lindsley JE (1998) Pre-steady-state analysis of ATP hydrolysis by Saccharomyces cerevisiae DNA topoisomerase II. 1. A DNA-dependent burst in ATP hydrolysis. Biochemistry 37: 7292-7298.

59. Schmidt BH, Osheroff N, Berger JM (2012) Structure of a topoisomerase II-DNA-nucleotide complex reveals a new control mechanism for ATPase activity. Nature Structural & Molecular Biology 19: 1147-1154.

60. Mueller-Planitz F, Herschlag D (2008) Coupling between ATP binding and DNA cleavage by DNA topoisomerase II - A unifying kinetic and structural mechanism. Journal of Biological Chemistry 283: 17463-17476.

61. Hammonds TR, Maxwell A (1997) The DNA dependence of the ATPase activity of human DNA topoisomerase II alpha. Journal of Biological Chemistry 272: 32696-32703.

62. Charvin G, Strick TR, Bensimon D, Croquette V (2005) Tracking topoisomerase activity at the single-molecule level. Annual Review of Biophysics and Biomolecular Structure. pp. 201-219.

63. Crisona NJ, Strick TR, Bensimon D, Croquette V, Cozzarelli NR (2000) Preferential relaxation of positively supercoiled DNA by E-coli topoisomerase IV in single-molecule and ensemble measurements. Genes & Development 14: 2881-2892.

64. Bray D (1992) Cell movements. New York: Garland Publishing.

65. Barnes S, Curtis H (1989) Biology, Fifth Edition. New York: Worth.

66. Longo G, Alonso-Sarduy L, Rio LM, Bizzini A, Trampuz A, et al. (2013) Rapid detection of bacterial resistance to antibiotics using AFM cantilevers as nano-mechanical sensors. Nature Nanotechnology 8: 522-526.

67. von Germar F, Barth A, Mäntele W (2000) Structural Changes of the Sarcoplasmic Reticulum Ca2+-ATPase upon Nucleotide Binding Studied by Fourier Transform Infrared Spectroscopy. Biophysical Journal 78: 1531-1540.

68. Krasteva M, Barth A (2007) Structures of the Ca2+–ATPase complexes with ATP, AMPPCP and AMPPNP. An FTIR study. Biochimica et Biophysica Acta (BBA) - Bioenergetics 1767: 114-123.

69. Chen W, Guidotti G (2001) Soluble apyrases release ADP during ATP hydrolysis. Biochemical and Biophysical Research Communications 282: 90-95.

70. Alonso-Sarduy L, Roduit C, Dietler G, Kasas S (2011) Human topoisomerase II-DNA interaction study by using atomic force microscopy. FEBS Lett 585: 3139-3145.

71. Hu B, Mayer MP, Tomita M (2006) Modeling Hsp70-mediated protein folding. Biophysical Journal 91: 496-507.

72. Tyagi NK, Fenton WA, Horwich AL (2009) GroEL/GroES cycling: ATP binds to an open ring before substrate protein favoring protein binding and production of the native state. Proceedings of the National Academy of Sciences of the United States of America 106: 20264-20269.
